# Army Legislation

**Published:** 1900-08

**Authors:** Rush S. Huidekoper

**Affiliations:** Secretary; Washington, D. C.


### CORRESPONDENCE.

### ARMY LEGISLATION.

COMMITTEE ON ARMY LEGISLATION,
P. O. BOX 189,
WASHINGTON, D. C., August 1, 1900.

DEAR COLLEAGUE: Senator Kenney's bill for the establishment of an organized Veterinary Corps in the United States Army passed the United States Senate as "Section 14 of S. 4300," A bill for the efficiency of the Army (known as the Army Reorganization Bill).

This Army Bill was under consideration for some ten days by the Military Committee of the House of Representatives, but action upon it has been delayed until the reconvening of Congress in December next.

If the present bill, "S. 4300," is then taken up, we feel assured of the passage of our veterinary section.

But we must foresee the probability of an entirely new Army Reorganization Bill being drafted to take its place, and in that case our section will again have to be acted upon in the Senate.

We have had the assurance of a majority of the Senators and of the Congressmen in the House of Representatives that they will support our bill.

This is not sufficient. We must have the promise of support from two-thirds to prevent the possibility of any accident or obstacle being placed in our way.

We have had to overcome the opposition of a few powerful individuals in the War Department and a few powerful Senators in the Military Committee of the Senate, who influenced the other members of the committee, even some of our friends.

Senator Gallinger said of the opposition of the War Department:

"Now, Mr. President, it seems to me that no valid objection has been made against this amendment. The Secretary of War, it is true, seems to have reported against it; but I take it that the Secretary of War knows less about this subject than General Sheridan, or General Miles, or the Quartermaster-General, or men who have been educated in military science know about it. Hence I certainly cannot lay as great stress upon his opinion as I lay upon the opinions of those great officers who were thoroughly acquainted with the needs of the Army and who speak from actual observation and experience."

The opposition advanced by members of the Military Committee of the Senate consisted of personal opinions. No argument of fact was advanced. On the contrary, facts were distorted, and such arguments were used as that: "The captain of a troop of cavalry knows more about a horse than any veterinarian from a college;" "it would pay the English Government to send over here and get forty or fifty or a hundred farriers to go down there [Africa] and save their horses now from the veterinarians who graduated from their colleges;" and a Senator who has a son in the army "would not see a veterinary surgeon rank with him."

An outcry was made against a "bureau," and a compromise was offered simply to give rank to the veterinarians employed in the cavalry.

Under the system of administration in the United States Army an efficient and economical service cannot be had in any other way than that provided for in our blil, any more than any business house could employ thirty or fifty workmen effectively without a foreman or superintendent.

Our opponents ignored entirely the service needed for the artillery, for the Quartermaster Department, for inspection of animal food, and for instruction in our military schools.

They ignored absolutely the experience of every other army in the world on this subject.

We can expect opposition again from some individuals. The printed data sent you during the last winter covers the subject.

For any misunderstanding of the subject, or argument in opposition we will, upon request from you, send at once the necessary facts in answer.

Here in Washington your Representatives are too busy to go into details, and leave matters to committees. Obtain their interest while they have time at home.

In the coming election remember: An old Representative is worth five new ones, if your friend; and is worse than ten new ones, if against you. Experience teaches them how to legislate. Remember also: A promise of "consideration," "careful consideration," etc., means absolutely nothing. Obtain a distinct promise of support, or continue until you do get it.

Interest the members of your State Legislature and obtain their influence to secure a fair hearing from your Senators.

We demand an organized veterinary corps, as provided for in S. 4300, when this bill passes, or as a part of the *first* Army Bill which does pass in its place when Congress reconvenes. Any compromise simply of rank, without organization, will not increase the efficiency of the army, and will set back the proper recognition of the profession a quarter of a century.

This is a question of merit, economy, and necessity for the efficiency of the army, and is not a political one, as the following names of some of our supporters show:

Senators: Allison, Iowa; Bacon, Georgia; Frye, Maine; Gallinger, New Hampshire; Hale, Maine; Kenney, Delaware; McEnery, Louisiana; Morgan, Alabama; Stewart, Nevada; and Wolcott, Colorado.

Congressmen: Allen, Mississippi; Capron, Rhode Island; Clayton, New York; Gaines, Tennessee; Hull, Iowa; Joy, Missouri; Lentz, Ohio; Mercer, Nebraska; Sulzer, New York; and Tawney, Minnesota.

In order that we may make an intelligent campaign before the reconvening of Congress, our State Secretaries in conjunction with the officers of the State veterinary associations will appoint committees and sub-committees for each State and Congressional district. You will be furnished with a list of Senators and Congressmen in your State, and you are begged to join with the committees in obtaining from them, when they have leisure at home, an interview to place the subject before them, and secure from them renewed promise of support for our bill next December.

Please inform your State Secretary *at once* if you will act on the committees; with your address, add county, or ward in cities.

At our annual meeting at Detroit in September we will complete our organization for canvass; should be positively assured of success, and know what opposition we must meet and overcome.

The eight thousand veterinarians in the United States to-day represent a profession, and are not to be classed on the pay-list with teamsters, as part of them are in the U. S. Army under the present system. For the committee,

RUSH S. HUIDEKOPER,
Secretary.

NOTICE. All veterinarians, whether members of the Association or not, are cordially invited and urged to attend the annual meeting at Detroit, Mich., September 4 to 6, 1900. This meeting will be the largest which has been held since the organization in 1863, and the scientific and practical papers promised will make it the most valuable and interesting. For information as to reduced railroad fares and special hotel rates, write your State Secretary, or the General Secretary, Dr. S. Stewart, Kansas City, Kansas.

LEONARD PEARSON,
President.

